# Burnout in ICUs in Portugal: is there? Are there differences between doctors and nurses?

**DOI:** 10.1186/cc9910

**Published:** 2011-03-11

**Authors:** C Teixeira, S Pereira, O Ribeiro, A Fonseca, A Carvalho

**Affiliations:** 1 Centro Hospitalar do Porto, Portugal; 2 Universidade dos Açores, Angra do Heroísmo, Portugal; 3 Faculdade de Medicina do Porto, Portugal; 4 Universidade Católica Portuguesa, Porto, Portugal

## Introduction

The aims were to identify whether there is burnout and the burnout levels of doctors and nurses working in ICUs (adult polyvalent units in northern Portugal). Also, to identify factors that may lead to the development of burnout in doctors and nurses working in the ICU.

## Methods

Application of a self-completion questionnaire with three items: the sociodemographic data of the study population, experiences in the workplace, and the Maslach Burnout Inventory - General Survey (Portuguese Version for Investigation 2006). For the implementation of methodological tools, we requested the authorization of the relevant institutional bodies, the ethics committee and directors of services. The professionals who participated in the study were asked for informed consent, whether formal or informal. Observation of the contexts of work and interviews was also done. In this study we will focus on the results of the questionnaire. Statistical analysis was performed using SPSS v.17.0.

## Results

A total of six hospitals, 10 polyvalent adult ICUs in the north of the country, 300 professionals, 73% nurses. Age of respondents was a median 32 years, with 8 years of professional experience and 4 years on the ICU. Results of the MBI: average levels of burnout in physicians and nurses working in the ICU. The risk of developing burnout is highest being a nurse 1:54 OR, yet there is no statistically significant difference at 95% (0.837, 2.834). Nine percent of professionals studied showed burnout, 31% with Burnout syndrome and high risk of burnout. Distribution of levels of burnout by occupational category: higher levels of emotional exhaustion in nurses, personal and professional achievement smaller in nurses, and higher depersonalization in doctors.

## Conclusions

The results of the study underline the importance of promoting the prevention of burnout in doctors and nurses in the ICU.
